# Establishment and validation of a risk scoring model for predicting the risk of bladder stones in patients with benign prostatic hyperplasia

**DOI:** 10.3389/fmed.2026.1795565

**Published:** 2026-04-16

**Authors:** Yuli Luo, Yi Gao, Hongzhi Fang, Changjian Shi, Jie Xu, Xinyi Li, Yunfei Li

**Affiliations:** Department of Urology, Renmin Hospital, Hubei University of Medicine, Shiyan, Hubei, China

**Keywords:** benign prostatic hyperplasia, bladder stones, nomogram, predictive model, risk factors

## Abstract

**Purpose:**

The aim of this study is to identify independent risk factors for bladder stones in patients with benign prostatic hyperplasia (BPH), and to develop and validate a nomogram prediction model for assessing the risk of bladder stones in this patient population.

**Methods:**

Retrospective analysis included 446 BPH patients (106 with bladder stones, 340 without) who underwent transurethral resection of the prostate (2023–2025). Univariate ROC (receiver operating characteristic), correlation analysis, LASSO regression, and multivariate logistic regression were used for variable screening and model construction. The cohort was split into training (70%) and validation (30%) sets. Model performance was evaluated via AUC (discrimination), calibration curves, Hosmer-Lemeshow test (calibration), and DCA (clinical utility).

**Results:**

Seven independent risk factors were identified: age, IPSS (International Prostate Symptom Score), serum uric acid, IPP (intravesical prostatic protrusion), PUA (prostatic urethral angle), TPV (total prostate volume), and urinary red blood cell count. The nomogram showed excellent discrimination (AUC: 0.865 in training set, 0.882 in validation set), good calibration (*p* > 0.05), and robust clinical utility.

**Conclusion:**

The nomogram overcomes univariate and multicollinearity limitations, enabling precise individualized risk assessment of bladder stones in BPH patients. It serves as a reliable tool for clinicians to guide personalized monitoring and prevention, potentially reducing incidence and healthcare burdens.

## 1 Introduction

Benign prostatic hyperplasia (BPH) is a common urological condition that virtually every aging man will experience. Prevalence rates are very high in older men, with 50 to 75% of men over 50 years and more than 70% of men over 70 years having some anatomical evidence of BPH. More than 80% of men over 70 years have some histological evidence of benign prostatic hyperplasia (1, 2). BPH often develops with various other complications, including acute urinary retention, hematuria, urinary tract infections, and urinary stones (3, 4). The most common symptoms in men with a diagnosis of benign prostatic hyperplasia are lower urinary tract symptoms (LUTS), which include voiding difficulties, such as weak stream and hesitancy, and the feeling of the bladder not emptying completely. These show a prevalence of 25 to 50% in men with BPH. These symptoms primarily arise from bladder outlet obstruction (BOO) induced by prostatic enlargement (5). Bladder stones represent a frequent manifestation of lower urinary tract calculi. Although they account for approximately 5% of all urinary stone diseases, they are responsible for a disproportionate 8% of urinary stone-related mortality (6). Bladder stones commonly occur in elderly males with lower urinary tract obstruction, such as benign prostatic hyperplasia (7, 8). More than half of patients diagnosed with bladder stones present with a constellation of symptoms, which may include suprapubic pain, dysuria (painful or difficult urination), hematuria (blood in the urine), urinary intermittency, increased urinary frequency, urgency, and generalized discomfort (9). The coexistence of BPH and bladder stones significantly increases the risk of recurrent urinary tract infections. Furthermore, prolonged stone-induced irritation and chronic inflammation can trigger squamous metaplasia of the bladder mucosa, a premalignant change that carries a risk of progression to bladder cancer. Concurrently, the physical obstruction caused by stones at the bladder neck or posterior urethra can exacerbate pre-existing BOO. This aggravated obstruction poses a threat to upper urinary tract integrity, potentially resulting in ureteral dilation, hydronephrosis, and ultimately, renal impairment (10). Current clinical guidelines recommend simultaneous surgical treatment of BPH and bladder stones; however, such patients are usually old with several comorbid conditions (11, 12). Given this demographic reality, coupled with increased operative time for combined procedures, perioperative risks are elevated, thereby underlining the importance of a procedure-sparing approach. The exact pathophysiological mechanisms linking BPH and the development of bladder stones remain obscure. Previous studies have identified age, PUA, IPP, TPV, transitional zone volume (TZV), gout, IPSS, and hypertension as potential risk factors for bladder stone formation in patients with BPH (13–16). Knowledge of these factors is important because it allows for the identification of high-risk patients and appropriate clinical management, which may include effective primary prevention. Current risk assessment for bladder stones in BPH patients has two key limitations: univariate prediction with low AUC values fails to meet clinical needs, and multicollinearity among variables introduces bias and instability into conventional multivariate regression, impairing model reliability and generalizability. This study analyzed clinical data from patients with BPH, and independent risk factors were ultimately identified and integrated into a practical, internally validated visual nomogram. This model provides a scientific basis for early risk stratification and targeted prevention of bladder stones in patients with benign prostatic hyperplasia.

## 2 Materials and methods

### 2.1 Patients

A retrospective analysis was performed on clinical data from patients who underwent transurethral resection of the prostate (TURP) at Shiyan People’s Hospital from 01/01/2023 to 31/10/2025. Patients were included if they were diagnosed with BPH in accordance with the Chinese Guidelines for Diagnosis and Treatment of Urological and Andrological Diseases (2019 edition) and scheduled for transurethral resection of the prostate (17), had a preoperative IPSS score greater than 7, and received postoperative pathological biopsy confirming benign prostatic hyperplasia. Patients were excluded from the analysis if they had a prior history of urinary tract stones, previous prostate surgery, a confirmed diagnosis of urinary tract tumors, neurogenic bladder, bladder foreign bodies, bladder diverticulum, bladder neck contracture, urethral stricture, or hydronephrosis, or presented with incomplete clinical data. Following application of the above eligibility criteria, 82 patients were excluded from the initial cohort, resulting in a final study population of 446 patients with BPH. According to the presence or absence of bladder stones, various subpopulations of the study population were generated. A stone group and a stone-free control group resulted from this classification: *n* = 106 and *n* = 340, respectively. All patients gave informed consent. The study was approved by the Ethics Committee of Shiyan People’s Hospital Affiliated to Hubei Medical College, and it was conducted according to the 1964 Declaration of Helsinki and its later amendments.

### 2.2 Variables for data collection and recording

We systematically identified 24 potential risk factors for concomitant bladder stones through a comprehensive review of relevant literature and an analysis of clinical records from our institution. These included patient age, body mass index (BMI), disease duration, serum uric acid, serum calcium, albumin, urinary leukocyte count, urinary erythrocyte count, urinary ketone body count, urinary glucose, urinary protein, and history of hypertension, coronary heart disease, and diabetes, as well as tPSA, IPSS score, and data from transabdominal ultrasound examinations. A single examiner obtained the prostate’s transverse, anteroposterior, and longitudinal dimensions via transabdominal ultrasonography in patients positioned supine, under standardized conditions of bladder filling (150–200 mL). The total prostate volume (TPV) was calculated using the corresponding formula. Transrectal Ultrasound (TRUS) Measurement Method: The maximum transverse, anteroposterior, and superior–inferior diameters of the prostate were measured on sagittal and transverse images. The TPV was then calculated using the ellipsoid formula: TPV = *π*/6 × transverse diameter × anteroposterior diameter × superior–inferior diameter. TZV: On TRUS images, the transitional zone of the prostate (typically located around the urethra) was identified, and its transverse, superior–inferior, and anteroposterior diameters were measured. The method for calculating TPV and TZV using transabdominal ultrasound is similar to that of TRUS; however, measurement accuracy may be slightly lower due to interference from the abdominal wall. The transitional zone index (TZI) was defined as TZI = transitional zone volume / total prostate volume. In addition, the following parameters were measured: IPP, PUA, maximum diameter and number of bladder stones, and post-void residual urine volume (PVR).

### 2.3 Statistical analysis

Categorical data are reported using percentage values, while continuous data are summarized by means of median (range) or the combination of mean and standard deviation (mean ± SD). Differences in baseline continuous variables between groups were assessed using independent samples Student’s *t*-test (for normally distributed data with homogeneous variance) or Wilcoxon rank-sum test (Mann–Whitney *U* test, for non-normally distributed data or heterogeneous variance). ROC curves were initially constructed for each candidate variable. The AUC was then computed to evaluate the discriminative ability of individual variables in predicting bladder stones. Variables with AUC > 0.5 were retained for subsequent analysis. Pearson correlation coefficients were calculated among candidate variables, and a correlation heatmap was generated to visualize inter-variable relationships. LASSO regression was employed to screen candidate variables. Variables selected by LASSO were incorporated into a multivariate logistic regression model to calculate odds ratios (OR) and 95% confidence intervals (CI), thereby identifying independent risk factors (*p* < 0.05). To achieve a sound development and evaluation of the model, the whole cohort was randomly divided into a training set and an independent validation set at a 7:3 ratio. The predictive nomogram was then constructed based on the independent risk factors identified using the training set and was applied using R software. Each variable was assigned points proportional to its regression coefficient in the multivariable model. These points were summed to derive a total score for each individual that corresponds to the predicted probability of bladder stone formation. The discriminative performance of the nomogram was assessed in the training and validation cohorts by plotting ROC curves and calculating the AUC. The AUC is an integrated measure quantifying the discriminatory ability of the model to differentiate patients with bladder stones from those without; hence, it reflects the model’s overall sensitivity and specificity. Calibration (defined as the agreement between predicted probabilities and observed outcomes) was evaluated using calibration plots for both the training and validation cohorts. In addition, decision curve analysis was applied to the model using net benefits over a range of clinically plausible probability cutoffs to test the model’s clinical applicability. All statistical analyses were performed using SPSS software version 26.0 (IBM Corp., Armonk, NY, United States). Based on the identified independent factors, the ROC curve was constructed using R software version 4.5.2 (R Foundation for Statistical Computing, Vienna, Austria).

## 3 Results

### 3.1 Clinical characteristics of study subjects

Flowchart of patient enrollment and group allocation (Supplementary Figure 1). Between January 2023 and October 2025, 446 consecutive patients diagnosed with BPH were stratified into two cohorts according to the presence of concomitant bladder stones. This resulted in a bladder stone cohort (Group 1, n = 106) and a non-stone cohort (Group 2, *n* = 340). A comparative analysis of the baseline clinical characteristics between these cohorts is presented in Table 1. There were statistically significant differences between the two groups in terms of age, BMI, diabetes, hypertension, IPSS, serum uric acid, serum creatinine, urinary leukocytes, urinary erythrocytes, urinary protein, IPP, PUA, TZI (all *p* < 0.05). while no statistically significant differences were observed in disease duration, history of coronary heart disease, history of acute urinary retention, tPSA, serum calcium, albumin, urinary ketones, urinary glucose, PVR, TZV (all *p* > 0.05). Additionally, based on transabdominal ultrasound findings, the maximum diameter of bladder stones ranged from 0.6 to 4.9 cm (median 1.30 cm, interquartile range 2.50 cm), with 80.37% of patients presenting with solitary stones.

**Table 1:** Clinical characteristics of the BPH group with concurrent bladder stones compared to the group without bladder stones.

Variables	Total (*n* = 446)	1 (*n* = 106)	2 (*n* = 340)	*p*
Age, Median (Q1, Q3)	69 (63, 75)	73.5 (69, 77)	68 (62, 73)	<0.001
BMI, Median (Q1, Q3)	23.44 (21.41, 25.5)	24.22(21.76,26.17)	23.32(21.34, 25.33)	0.03
Time, Median (Q1, Q3)	36 (12, 60)	26 (12, 60)	36 (12, 60)	0.392
Diabetes, *n* (%)				0.002
No	374 (84)	78 (74)	296 (87)	
Yes	72 (16)	28 (26)	44 (13)	
Hypertension, *n* (%)				0.003
No	279 (63)	53 (50)	226 (66)	
Yes	167 (37)	53 (50)	114 (34)	
Coronary heart disease, *n* (%)				0.954
No	380 (85)	91 (86)	289 (85)	
Yes	66 (15)	15 (14)	51 (15)	
Acute urinary retention, *n* (%)				0.16
No	275 (62)	72 (68)	203 (60)	
Yes	171 (38)	34 (32)	137 (40)	
tPSA, Median (Q1, Q3)	3.05 (1.45, 5.22)	2.39 (1.1, 4.84)	3.22 (1.55, 5.36)	0.057
IPSS, Median (Q1, Q3)	18 (15.25, 22)	20 (17, 23)	18 (15, 22)	0.01
Serum uric acid, Median (Q1, Q3)	304.85(260.1,360.87)	318.3(267.47,385.5)	299.15(254, 356.6)	0.006
Serum calcium, Median (Q1, Q3)	2.35 (2.28, 2.41)	2.34 (2.27, 2.42)	2.35 (2.28, 2.41)	0.798
Albumin, Median (Q1, Q3)	42.4 (39.7, 45.18)	42.2 (39.92, 44.7)	42.45 (39.58, 45.3)	0.674
Creatinine, Median (Q1, Q3)	77.45 (68.62, 88.9)	81 (71.93, 91.07)	76.3 (68.18, 87.8)	0.033
Urinary white blood cells, *n* (%)				<0.001
Count < 2+	351 (79)	67 (63)	284 (84)	
Count ≥ 2+	95 (21)	39 (37)	56 (16)	
Urinary red blood cells, *n* (%)				<0.001
Count < 2+	297 (67)	52 (49)	245 (72)	
Count ≥ 2+	149 (33)	54 (51)	95 (28)	
Ketone bodies in urine, *n* (%)				0.056
Count < 2+	426 (96)	105 (99)	321 (94)	
Count ≥ 2+	20 (4)	1 (1)	19 (6)	
Glucose in urine, *n* (%)				0.708
Count < 2+	424 (95)	102 (96)	322 (95)	
Count ≥ 2+	22 (5)	4 (4)	18 (5)	
Protein in urine, *n* (%)				0.01
Count <2 +	395 (89)	86 (81)	309 (91)	
Count ≥ 2+	51 (11)	20 (19)	31 (9)	
IPP, Median (Q1, Q3)	7.62 (4.19, 11.62)	8.44 (4.66, 16.24)	7.38 (3.96, 10.53)	0.006
PVR, Median (Q1, Q3)	0 (0, 80)	0 (0, 45)	0 (0, 80)	0.118
TPV, Median (Q1, Q3)	52.01 (37.2, 72.59)	47.25(35.66,66.46)	52.89(37.39,74.27)	0.102
PUA, Mean±SD	28.92 ± 8.32	33.67 ± 7.23	27.44 ± 8.09	< 0.001
TZV, Median (Q1, Q3)	27.95(18.54, 42.28)	27.08(20.43,41.98)	28.15 (18.09, 42.2)	0.906
TZI, Mean±SD	0.54 ± 0.12	0.57 ± 0.11	0.53 ± 0.13	< 0.001

BPH, benign prostatic hyperplasia; BMI, Body Mass Index; tPSA, prostatic protrusion; PVR, Post-void Residual; TPV, total prostate volume; PUA, prostatic urethral angel; TZV, transitional zone volume; TZI, transition zone index.

### 3.2 Results of univariate ROC analysis

**Figure 1:**
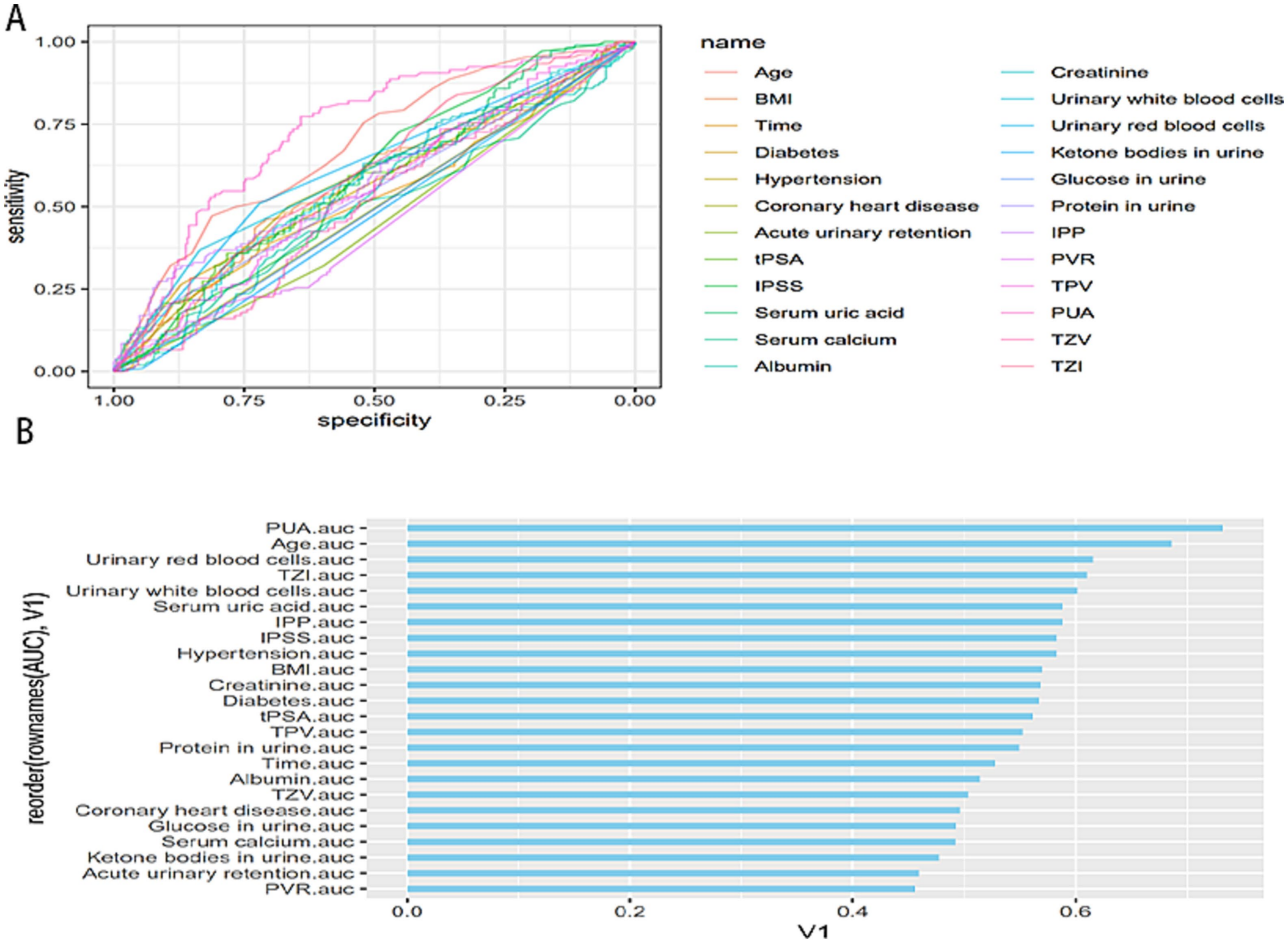
**(A)** ROC curves for predicting bladder stones in patients with BPH based on various clinical variables. The horizontal axis represents specificity, and the vertical axis represents sensitivity. Curves closer to the upper left corner indicate better predictive performance; **(B)** AUC value ranking bar chart for univariate ROC curves. The horizontal axis represents AUC values, with higher values indicating stronger predictive performance; variables are sorted from highest to lowest AUC value.

Univariate ROC analysis showed that various clinical factors had different predictive values for bladder stones in patients with BPH (Figure 1A). PUA exhibited the highest AUC value (Figure 1B), followed by age, urinary erythrocytes, TZI, urinary leukocytes, and serum uric acid. All these variables showed moderate predictive efficacy, with none exceeding an AUC of 0.8, suggesting limited predictive value when used alone. These findings support the need for a combined predictive model incorporating multiple variables to improve diagnostic accuracy.

### 3.3 Correlation heatmap analysis results

To clarify the strength of the associations and degree of collinearity between clinical variables, Pearson correlation tests were performed on the included variables, and a heatmap was generated (Supplementary Figure 2). The inter-variable correlation analysis demonstrated a spectrum of associations within which some pairs showed significant multicollinearity, characterized by an absolute correlation coefficient |*r*| > 0.7. Therefore, before the actual modeling, a stringent variable selection procedure was followed to allow for collinearity and model overfitting, which could yield biased parameter estimation. First, strong correlation between TPV and TZV (*r* > 0.8) showed that serious multicollinearity exists, which could bias their coefficients if both are included in a single multivariate regression model. Second, moderate correlations between TZI and both TPV and TZV must be considered when selecting variables. All these results are typical of the complex and frequently collinear nature of the relationships among the examined clinical variables. Therefore, conventional multivariate regression techniques are less frequently used because they do not always address the problems of multicollinearity, which can destabilize model estimates and obscure the effects of real predictors. Thus, using LASSO regression was a robust alternative to simultaneous dimensionality reduction and variable selection. It is an approach that aims to improve the stability of models through penalization while enhancing the reliability of specific predictor identification.

### 3.4 LASSO regression variable selection

**Figure 2:**
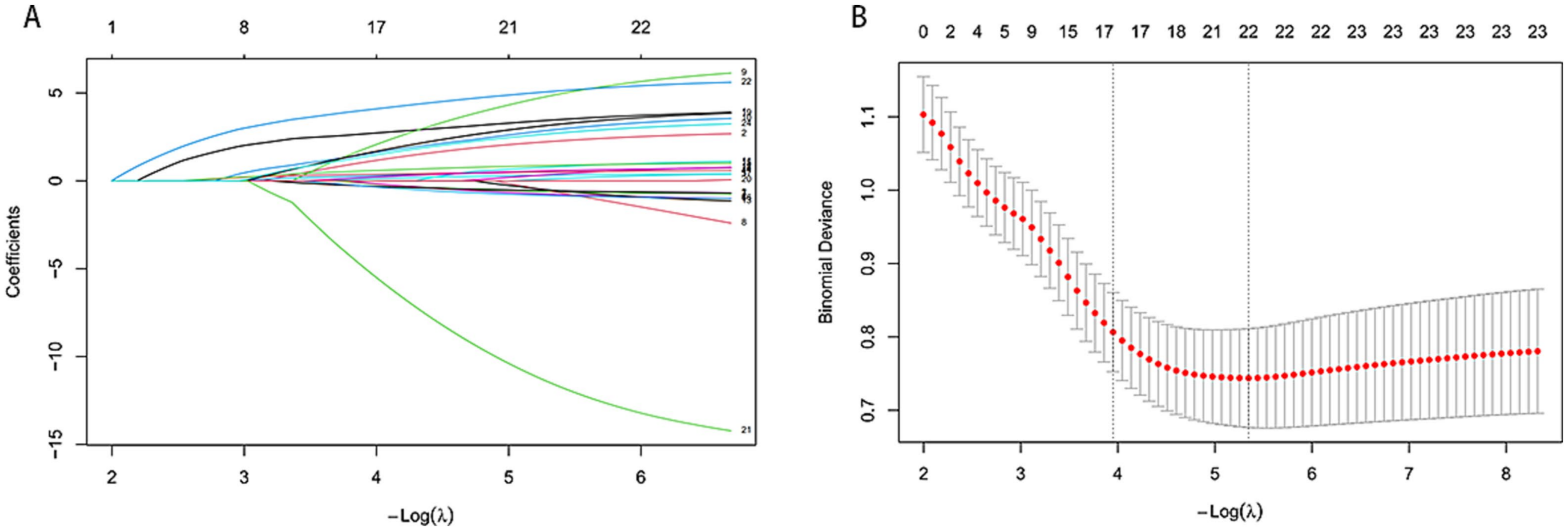
**(A)** LASSO regression coefficient profile; **(B)** LASSO regression cross-validation error plot.

To mitigate multicollinearity between variables and screen to find the optimal predictive factors, variables demonstrating an area under the curve (AUC) greater than 0.5 in univariate receiver operating characteristic (ROC) analysis were included in the least absolute shrinkage and selection operator (LASSO) regression. The optimal regularization parameter *λ* was identified using 10-fold cross-validation, balancing the model’s predictive accuracy and parsimony (Figure 2A). One panel shows the LASSO regression coefficient contour plot; as the −Log(*λ*) value corresponding to the regularization parameter *λ* increases, the regularization strength increases—the variable coefficients gradually shrink toward zero, and redundant variables are eliminated (Figure 2B). Two key reference lines on the cross-validation curves are *λ*.min, the *λ* value at which the minimum mean cross-validated error occurred, and *λ*.1se, the *λ* value at which the minimum mean cross-validated error occurred within one standard error of that for *λ*.min. The model with the best predictive accuracy was therefore determined using the λ.min criterion, while the more parsimonious model was derived from the λ.1se criterion, which favored simplicity at a slight trade-off in predictive error. Based on the selection results from λ.min, 12 candidate variables with non-zero coefficients were ultimately retained: age, BMI, hypertension, diabetes, serum uric acid, IPSS, urinary leukocytes, urinary erythrocytes, urinary protein, IPP, PUA, and TZI.

### 3.5 Establishing a line chart model

**Figure 3:**
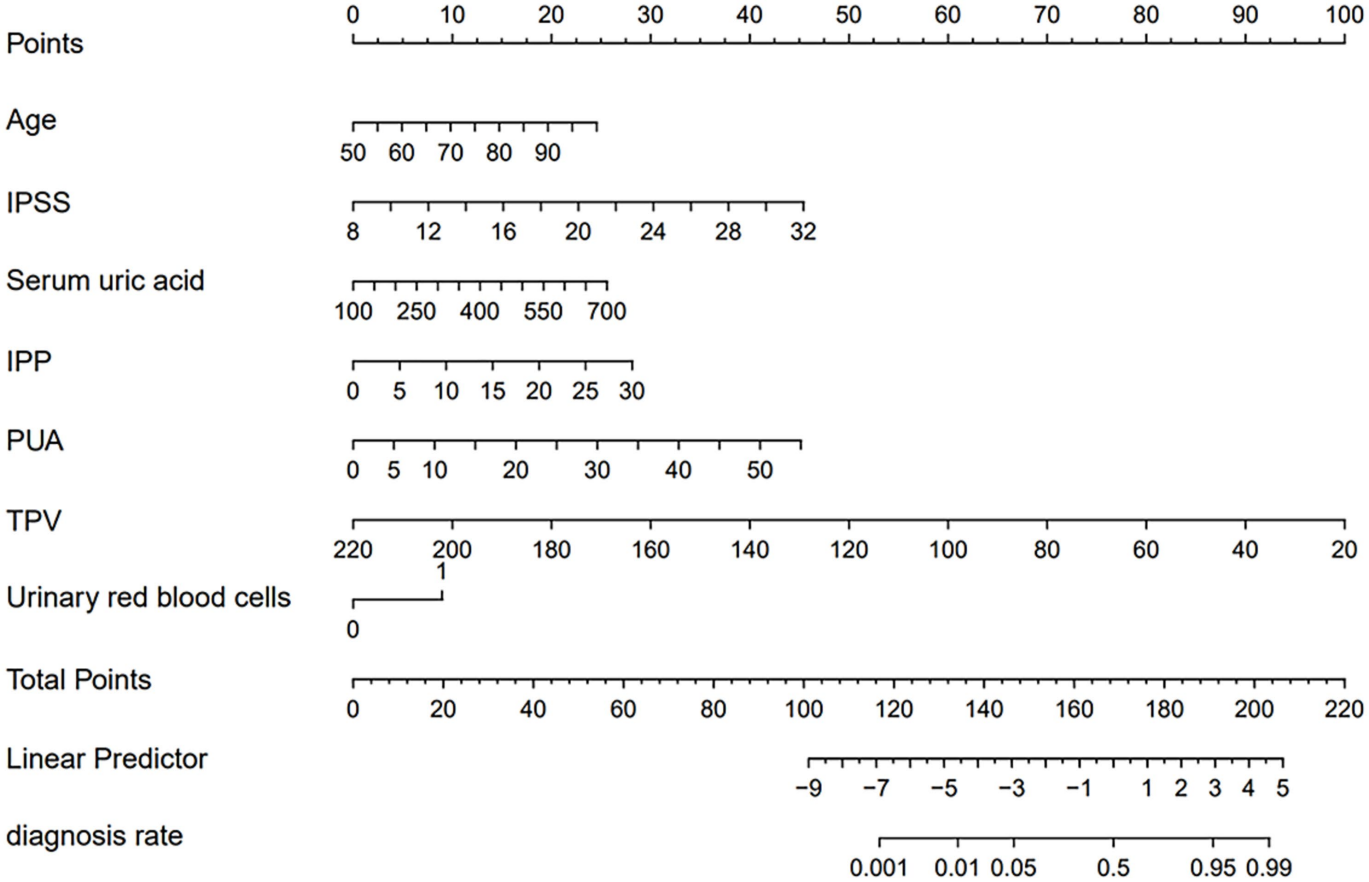
Nomogram prediction model. Points: Individual scores for each risk factor; Age: Age (years); Serum uric acid: Serum uric acid (μmol/L); IPSS: International Prostate Symptom Score; Urinary red blood cells: Urinary red blood cells (0 = <2+, 1 = ≥2+); IPP: Intravesical Prostatic Protrusion (mm); PUA: Prostatic Urethral Angle (degrees); TPV: Total Prostatic Volume (ml); Total Points: Sum of individual scores for 7 variables; Linear Predictor: Linear predicted value; Diagnosis Rate: Predicted probability of concomitant bladder stones.

The 12 candidate variables filtered through LASSO regression were included in the multivariate logistic regression analysis. Considering “the presence of concurrent bladder stones” as the outcome variable, the independent risk factors were determined by stepwise regression (inclusion criterion *p* < 0.05; exclusion criterion *p* > 0.10). Eventually, seven independent risk factors that had robust relationships with regard to bladder stones among BPH patients were found and used to construct the nomogram (Figure 3). This nomogram contains four major modules: “Score Axis,” “Variable Value Axis,” “Total Score Axis,” and “Predicted Probability Axis.” Each independent risk factor has its dedicated value axis from which the corresponding scores can be read on the “Score Axis” according to its real value. The individual scores for the seven variables are summed to obtain the total score (range: 0–220 points). Finally, the patient’s individualized risk of developing bladder stones is determined by reading the corresponding predicted probability (range 0.001–0.99) on the “Predicted Probability Axis” based on the total score. Example: Age 70 corresponds to 30 points, serum uric acid 350 μmol/L corresponds to 25 points, IPSS 20 corresponds to 20 points, urine red blood cells ≥2+ corresponds to 25 points, IPP 10 mm corresponds to 35 points, PUA 35 degrees corresponds to 30 points, TPV 60 mL corresponds to 25 points. Total score: 190 points. Predicted probability of developing bladder stones: approximately 0.95.

### 3.6 Validation of the line chart model

**Figure 4:**
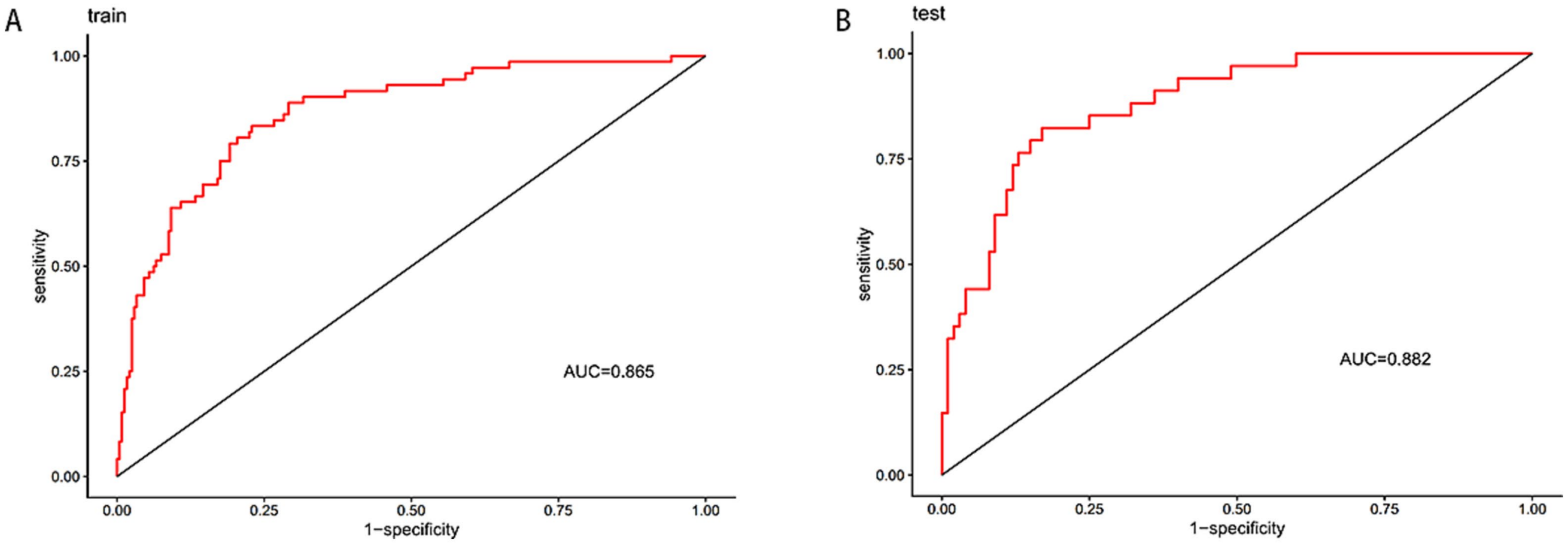
Receiver operating characteristic curves of the nomogram in the training set **(A)** and test set **(B)** groups.

**Figure 5:**
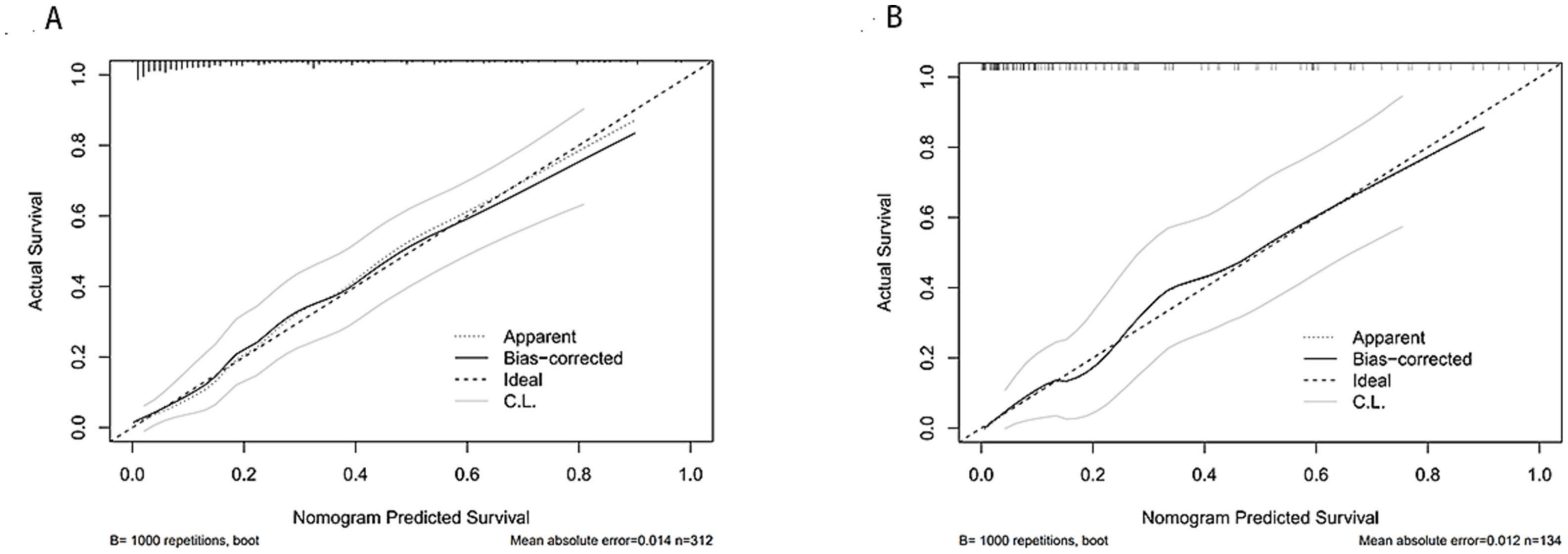
**(A)** Training set calibration curves; **(B)** test set calibration curves.

**Figure 6:**
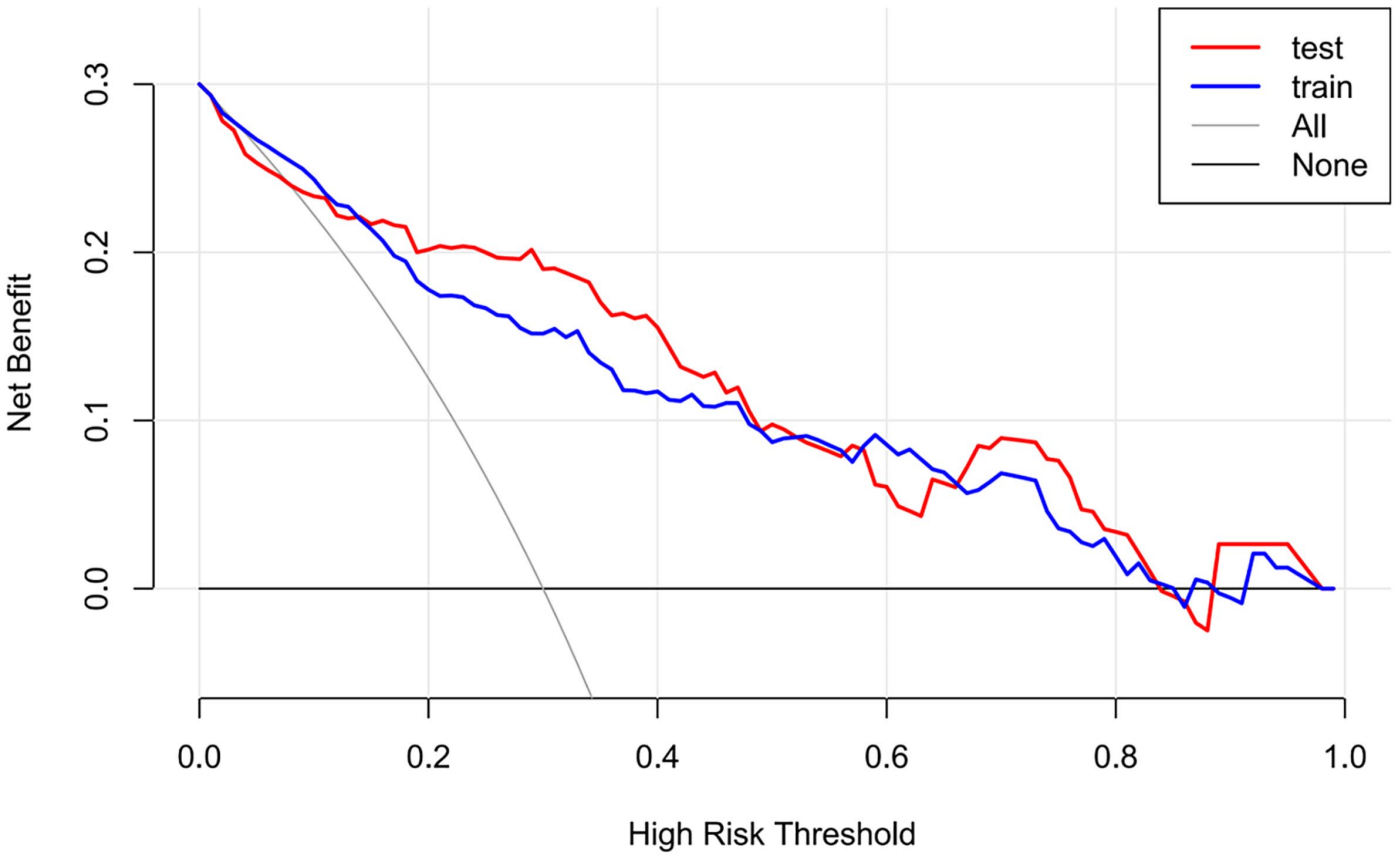
Clinical decision curves of the model. The horizontal axis represents the probability of high-risk thresholds, while the vertical axis denotes net clinical benefit. The train curve reflects the training set model, the test curve represents the validation set model, all indicates the “treat all” strategy, and none denotes the “do not treat any” strategy.

A dual validation strategy was performed with the training and validation sets, indeed showing the predictive performance of the nomogram model in three dimensions: discriminative power, calibration, and clinical usefulness. The training set ROC curve showed an AUC of 0.865 (95% CI: 0.8167–0.9128), as shown in Figure 4A; the validation set AUC was 0.882 (95%CI: 0.8197–0.9432); the data are shown in Figure 4B. Therefore, the model performed well, which indicates excellent internal validation and discriminative power to distinguish effectively between high-risk and low-risk patients. The calibration curve for the training set showed that the bias-adjusted model curve was close to the ideal calibration line; MAE = 0.014 (*n* = 312,1,000 Bootstrap resamples) (Figure 5A); in the calibration curve for validation, it is very well fit, for the bias-corrected curve follows closely the ideal calibration line producing MAE = 0.012 (*n* = 134,1,000 Bootstrap resamples) (Figure 5B). This shows that the model produces almost no differences between predicted risks and actual patient outcomes; thus, it is well calibrated and can quantify risk with excellent precision. The clinical decision curve shows that, in the threshold probability of 0.0 to 0.8, both the training and validation set nomogram curves are above the “treat all” and “do not treat all” curves (Figure 6). Within this threshold interval, there is a considerable net benefit when intervening in clinical practice based on model predictions. Highly valuable in preventing overtreatment or missed diagnosis, this model provides significant practical value in clinical decision-making. In general, this newly developed nomogram model has been extensively validated by various forms of validation and has successfully predicted the clinical risk of bladder stone formation in patients with BPH.

## 4 Discussion

Concomitant bladder stones complicate BPH management, and accurate personalized risk stratification is critical for timely intervention. However, robust predictive tools remain underdeveloped. This study addresses this gap by developing a validated visual nomogram to predict bladder stone risk in BPH patients using advanced variable selection and model-building techniques, facilitating high-risk patient identification and personalized intervention. BPH manifests in several different ways, but lower urinary tract symptoms—urinary frequency, urgency, a poor stream, and nocturia—are the hallmark symptoms (18). While these symptoms impose a direct physical burden, they are also increasingly affecting the quality of life of the patients who suffer from them. The pathogenesis of bladder stones involves multiple factors, with BOO caused by benign prostatic hyperplasia being the primary cause (7). This direct obstruction can lead to urinary dysfunction and urinary retention, thereby creating a microenvironment conducive to stone nucleation and growth (19). Therefore, early screening and risk stratification for bladder stone formation in patients with benign prostatic hyperplasia, coupled with targeted preventive measures, are crucial for improving patient outcomes.

The role of ultrasound parameters (such as IPP, PUA, and TPV) in predicting the clinical progression of prostate cancer is receiving increasing attention. Previous findings suggesting that IPP, PUA, and TPV serve as effective surrogate markers for BOO have been confirmed (20). The term “IPP” refers to a pathological condition in which prostate tissue enlarges and protrudes into the bladder lumen. This compresses the bladder neck, disrupts its normal funnel-shaped voiding mechanism, and ultimately leads to urinary retention and bladder stone formation (15). IPP exhibits a positive correlation with the severity of bladder outlet obstruction (21); There is a positive correlation between IPP and the severity of bladder outlet obstruction; previous studies have confirmed that IPP can predict complications associated with BPH (22, 23). This study indicates that IPP is an independent risk factor for the development of bladder stones. The adjusted odds ratio (OR) for IPP was 1.133, indicating an approximately 13.3% increased risk of developing bladder stones. The PUA is the angle formed by the curvature of the prostatic urethra at the Verumontanum on a mid-sagittal ultrasound image. Studies have shown that the prostatic urethral angle is associated with the severity of BOO (24). The OR for PUA is 1.115. For patients with BPH, a 1-degree increase in PUA is associated with an 11.5% increase in the risk of bladder stone formation. Indeed, some studies have indicated that age is an independent risk factor for bladder stone formation (13, 25). With advancing age, intravesical pressure increases, while detrusor contractility and urinary efficiency decline. Changes in metabolic homeostasis and a weakening of the urinary tract epithelial defense mechanisms collectively create a pathological environment more conducive to the formation of crystals and stones. This study confirms that age is an independent risk factor for the development of bladder stones in patients with benign prostatic hyperplasia. The odds ratio for age was 1.067, meaning that for every additional year of age in patients with benign prostatic hyperplasia, the risk of developing bladder stones increases by 6.7%. This risk factor has become a classical one relating to metabolic disorders associated with the formation of stones: increased serum uric acid levels. Indeed, some studies indicate that men with bladder stones probably present higher concentrations of uric acid compared to men without bladder stones (19). Elevated serum uric acid levels have become a classic risk factor among the metabolic disorders associated with stone formation. In fact, some studies suggest that men with bladder stones may have higher uric acid concentrations than men without bladder stones (20). Hyperuricemia leads to the precipitation of uric acid and may combine with ionized calcium to form calcium urate crystals. Urinary stasis exacerbates this process, ultimately leading to the formation of macroscopic stones. Our findings quantitatively confirm this association. Serum uric acid was identified as a significant risk factor, with an odds ratio (OR) of 1.006. This implies that, within the BPH cohort studied, a 1 μmol/L increase in serum uric acid concentration is associated with an approximate 0.6% increase in the risk of bladder stone formation. Furthermore, the inclusion of the IPSS score, urinary red blood cells, and TPV further refined the risk assessment system. The IPSS score directly reflects the severity of lower urinary tract symptoms (26). Higher scores indicate more pronounced urinary retention and abnormal urodynamics caused by obstruction. Positive occult blood in urine correlates with bladder stones: first, friction from stones might injure the urinary tract mucosa and, thus, release blood; second, products like hemoglobin-shed from the injured urinary tract or other tissues-might serve as an organic matrix facilitating crystal formation and accelerating the growth of stones (27). Indeed, the presence of a red blood cell in a routine urine test at a level of 2+ or above would raise concerns about urinary tract stones (28). TPV increases mechanical obstruction at the bladder outlet, indirectly increasing the risk of stone formation (29). Therefore, these three elements broaden the field of risk prediction in symptomatic, pathological injury, and anatomical structural levels.

This study employed univariate ROC analysis to establish the predictive capacity of each index efficiently, while LASSO regression performed dimensionality reduction after the collinearity problems were visualized by means of correlation heatmaps. Eventually, a multivariable logistic regression showed seven independent risk factors that go as follows: age, serum uric acid, IPSS, urinary erythrocytes, IPP, PUA, and TPV to develop a nomogram model in this case. This approach will more appropriately correct for bias in standard regression variability and assure greater robustness concerning the results. The model demonstrates high discriminatory power (AUC for the training set = 0.865, 95% CI: 0.8167–0.9128; AUC for the validation set = 0.882, 95% CI: 0.8197–0.9432) and high accuracy (MAE for the training set = 0.014, MAE for the validation set = 0.012). Besides, there is strong clinical usefulness. A nomogram is a graphical representation of a complex regression equation; it is a scoring instrument that allows for easy visual calculations of total points concerning readily measurable variables at bedside care and does not demand extra burdens of tests. It could then serve as a quick tool to help derive such probabilistic risks in patients and, hence, facilitate the construction of intervention strategies based on intuitive evidence.

This helps physicians stratify risks for BPH patients: for the high-risk group, proactive intervention is to be considered by controlling serum uric acid levels, improving urinary function, treating urinary tract infections, and, when necessary, early prostate surgery to relieve obstruction and essentially reduce the risk of stone formation. More moderately lower-risk patients would have regular monitoring, and health advice would be given to avoid unnecessary treatments. This model provides much greater net clinical benefit than treating all or not treating all, at threshold probabilities of 0.0 to 0.8, according to DCA results. Its advantage is particularly pronounced within the clinically relevant threshold range of 0.1–0.6, confirming its efficacy in supporting clinical decision-making and reducing both patient and societal healthcare burdens. However, this study has several limitations that should be addressed. First, single-center and retrospective designs limit the diversity in patient cohorts and introduce selection bias. Therefore, the external validity and generalizability of the nomogram need to be validated in larger multicenter prospective cohorts. Second, some relevant confounders, such as detailed dietary patterns, specific medication histories, and lifestyle factors, were not considered in this analysis, which possibly may have affected the predictive accuracy and comprehensiveness of the models. Finally, the model presented is inherently cross-sectional and provides a static risk estimate at one point in time, which was unable to take into consideration the dynamic evolution of risk factors through time or even the course of stone risk that this would create over extended patient follow-up.

## 5 Conclusion

In summary, this study presents a nomogram model based on seven independent risk factors that accurately and conveniently provides an individualized prediction of the risk of bladder stone formation in patients with BPH. It demonstrates excellent discrimination, calibration, and clinical utility. This scientifically grounded risk assessment tool allows clinicians to identify high-risk patients early and develop intervention strategies tailored to individual needs. As such, it is important in reducing the incidence of bladder stones and improving patient outcomes.

## Data Availability

The original contributions presented in the study are included in the article/Supplementary material, further inquiries can be directed to the corresponding author.

## Supplementary material

SUPPLEMENTARY FIGURE 1 Flowchart of study participants.

SUPPLEMENTARY FIGURE 2 Heatmap of correlations among clinical variables, color intensity and numerical values indicate correlation coefficient magnitude; red denotes positive correlation, blue denotes negative correlation; **P*<0.05, ***P*<0.01, ****P*<0.001; Correlation strength: |*r*| 0.1–0.3 indicates weak correlation, 0.3–0.5 indicates moderate correlation, >0.5 indicates strong correlation.
