# Supplementary material for: Establishment and validation of a risk scoring model for predicting the risk of bladder stones in patients with benign prostatic hyperplasia
Source: Front Med (Lausanne). 2026 Apr 16;13:1795565. doi: 10.3389/fmed.2026.1795565 (PMC13128432; doi:10.3389/fmed.2026.1795565)
Supplement: Supplementary file 1 [file Table_1.docx]

| Variables | Total (n = 446) | 1 (n = 106) | 2 (n = 340) | p |
| --- | --- | --- | --- | --- |
| Age, Median (Q1,Q3) | 69 (63, 75) | 73.5 (69, 77) | 68 (62, 73) | < 0.001 |
| BMI, Median (Q1,Q3) | 23.44 (21.41, 25.5) | 24.22(21.76,26.17) | 23.32(21.34,25.33) | 0.03 |
| Time, Median (Q1,Q3) | 36 (12, 60) | 26 (12, 60) | 36 (12, 60) | 0.392 |
| Diabetes, n (%) |  |  |  | 0.002 |
| No | 374 (84) | 78 (74) | 296 (87) |  |
| Yes | 72 (16) | 28 (26) | 44 (13) |  |
| Hypertension, n (%) |  |  |  | 0.003 |
| No | 279 (63) | 53 (50) | 226 (66) |  |
| Yes | 167 (37) | 53 (50) | 114 (34) |  |
| Coronary heart disease, n (%) | |  |  | 0.954 |
| No | 380 (85) | 91 (86) | 289 (85) |  |
| Yes | 66 (15) | 15 (14) | 51 (15) |  |
| Acute urinary retention, n (%) | |  |  | 0.16 |
| No | 275 (62) | 72 (68) | 203 (60) |  |
| Yes | 171 (38) | 34 (32) | 137 (40) |  |
| tPSA, Median (Q1,Q3) | 3.05 (1.45, 5.22) | 2.39 (1.1, 4.84) | 3.22 (1.55, 5.36) | 0.057 |
| IPSS, Median (Q1,Q3) | 18 (15.25, 22) | 20 (17, 23) | 18 (15, 22) | 0.01 |
| Serum uric acid, Median (Q1,Q3) | 304.85(260.1,360.87) | 318.3(267.47,385.5) | 299.15(254, 356.6) | 0.006 |
| Serum calcium, Median (Q1,Q3) | 2.35 (2.28, 2.41) | 2.34 (2.27, 2.42) | 2.35 (2.28, 2.41) | 0.798 |
| Albumin, Median (Q1,Q3) | 42.4 (39.7, 45.18) | 42.2 (39.92, 44.7) | 42.45 (39.58, 45.3) | 0.674 |
| Creatinine, Median (Q1,Q3) | 77.45 (68.62, 88.9) | 81 (71.93, 91.07) | 76.3 (68.18, 87.8) | 0.033 |
| Urinary white blood cells, n (%) | |  |  | < 0.001 |
| Count<2+ | 351 (79) | 67 (63) | 284 (84) |  |
| Count≥2+ | 95 (21) | 39 (37) | 56 (16) |  |
| Urinary red blood cells, n (%) |  |  |  | < 0.001 |
| Count<2+ | 297 (67) | 52 (49) | 245 (72) |  |
| Count≥2+ | 149 (33) | 54 (51) | 95 (28) |  |
| Ketone bodies in urine, n (%) | |  |  | 0.056 |
| Count<2+ | 426 (96) | 105 (99) | 321 (94) |  |
| Count≥2+ | 20 (4) | 1 (1) | 19 (6) |  |
| Glucose in urine, n (%) |  |  |  | 0.708 |
| Count<2+ | 424 (95) | 102 (96) | 322 (95) |  |
| Count≥2+ | 22 (5) | 4 (4) | 18 (5) |  |
| Protein in urine, n (%) |  |  |  | 0.01 |
| Count<2+ | 395 (89) | 86 (81) | 309 (91) |  |
| Count≥2+ | 51 (11) | 20 (19) | 31 (9) |  |
| IPP, Median (Q1,Q3) | 7.62 (4.19, 11.62) | 8.44 (4.66, 16.24) | 7.38 (3.96, 10.53) | 0.006 |
| PVR, Median (Q1,Q3) | 0 (0, 80) | 0 (0, 45) | 0 (0, 80) | 0.118 |
| TPV, Median (Q1,Q3) | 52.01 (37.2, 72.59) | 47.25(35.66,66.46) | 52.89(37.39,74.27) | 0.102 |
| PUA, Mean±SD | 28.92 ± 8.32 | 33.67 ± 7.23 | 27.44 ± 8.09 | < 0.001 |
| TZV, Median (Q1,Q3) | 27.95(18.54, 42.28) | 27.08(20.43,41.98) | 28.15 (18.09, 42.2) | 0.906 |
| TZI, Mean±SD | 0.54 ± 0.12 | 0.57 ± 0.11 | 0.53 ± 0.13 | < 0.001 |
