# Supplementary figures and images for: Establishment and validation of a risk scoring model for predicting the risk of bladder stones in patients with benign prostatic hyperplasia
Source: Front Med (Lausanne). 2026 Apr 16;13:1795565. doi: 10.3389/fmed.2026.1795565 (PMC13128432; doi:10.3389/fmed.2026.1795565)

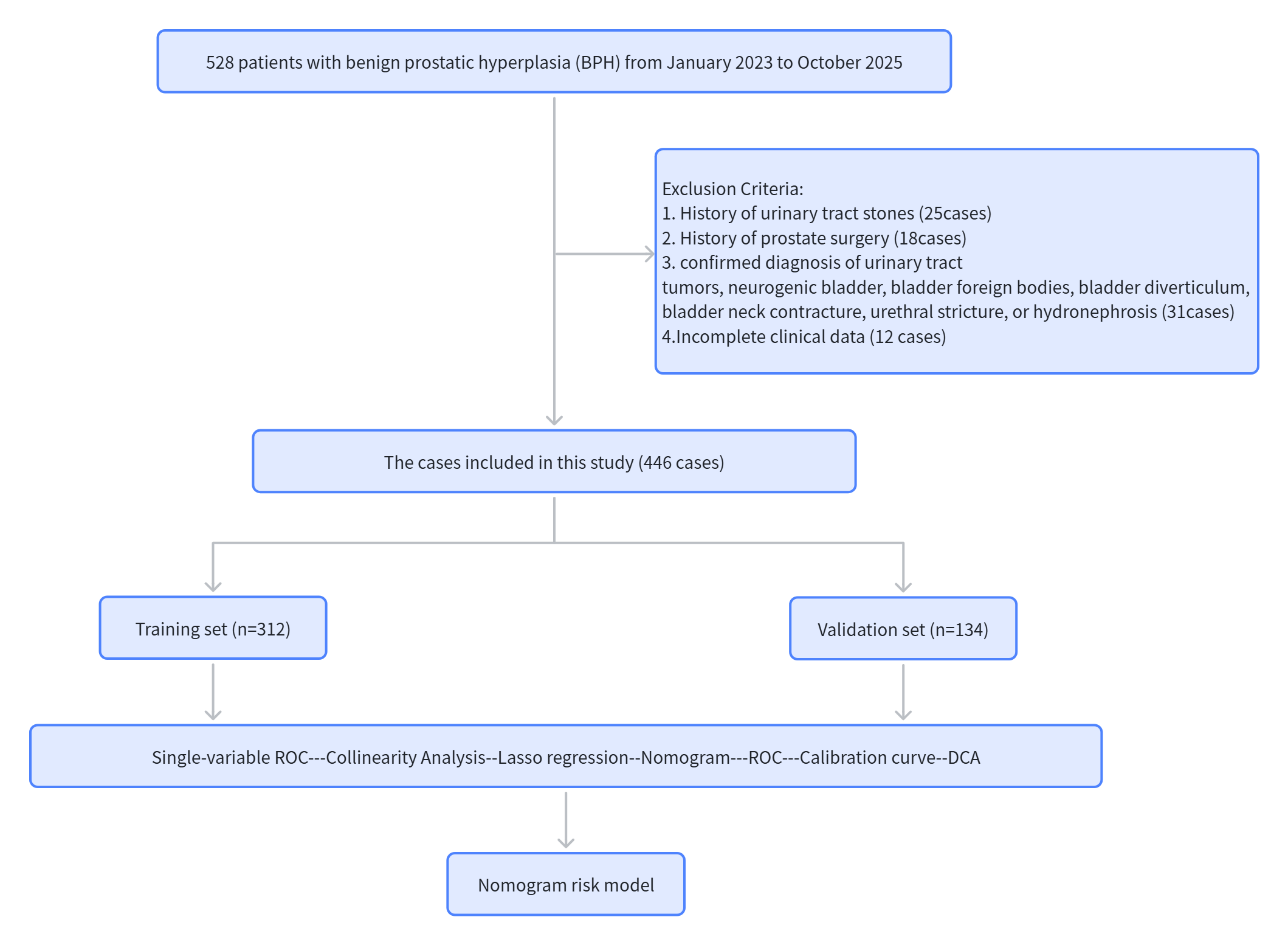

Supplement: SUPPLEMENTARY FIGURE 1 — Flowchart of study participants. [file Image_1.tif]

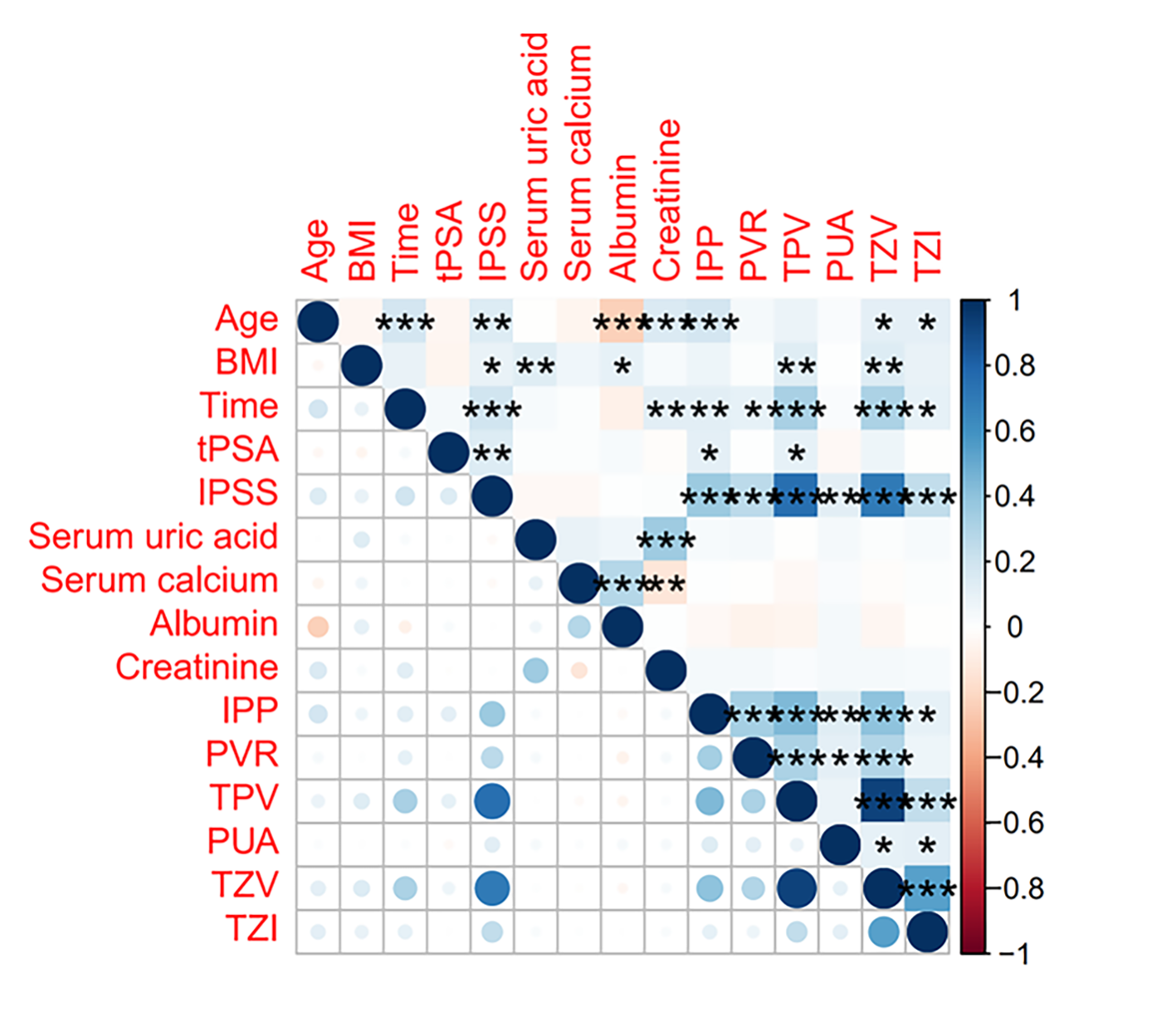

Supplement: SUPPLEMENTARY FIGURE 2 — Heatmap of correlations among clinical variables, color intensity and numerical values indicate correlation coefficient magnitude; red denotes positive correlation, blue denotes negative correlation; *P<0.05, **P<0.01, ***P<0.001; Correlation strength: |r| 0.1–0.3 indicates weak correlation, 0.3–0.5 indicates moderate correlation, >0.5 indicates strong correlation. [file Image_2.tif]
